# A degron-based approach to manipulate Eomes functions in the context of the developing mouse embryo

**DOI:** 10.1073/pnas.2311946120

**Published:** 2023-10-23

**Authors:** Alexandra M. Bisia, Ita Costello, Maria-Eleni Xypolita, Luke T. G. Harland, Philipp J. Kurbel, Elizabeth K. Bikoff, Elizabeth J. Robertson

**Affiliations:** ^a^Sir William Dunn School of Pathology, University of Oxford, Oxford OX1 3RE, United Kingdom

**Keywords:** Eomes, dTAG, degron, mouse, embryo

## Abstract

The T-box transcription factor Eomesodermin (Eomes), also known as Tbr2, plays essential roles in the early mouse embryo. Loss-of-function mutant embryos arrest at implantation due to Eomes requirements in the trophectoderm cell lineage. Slightly later, expression in the visceral endoderm promotes anterior visceral endoderm formation and anterior–posterior axis specification. Early induction in the epiblast beginning at day 6 is necessary for nascent mesoderm to undergo epithelial to mesenchymal transition (EMT). Eomes acts in a temporally and spatially restricted manner to sequentially specify the yolk sac haemogenic endothelium, cardiac mesoderm, definitive endoderm, and axial mesoderm progenitors during gastrulation. Little is known about the underlying molecular mechanisms governing Eomes actions during the formation of these distinct progenitor cell populations. Here, we introduced a degron-tag and mCherry reporter sequence into the Eomes locus. Our experiments analyzing homozygously tagged embryonic stem cells and embryos demonstrate that the degron-tagged Eomes protein is fully functional. dTAG (degradation fusion tag) treatment in vitro results in rapid protein degradation and recapitulates the Eomes-null phenotype. However in utero administration of dTAG resulted in variable and lineage-specific degradation, likely reflecting diverse cell type–specific Eomes expression dynamics. Finally, we demonstrate that Eomes protein rapidly recovers following dTAG wash-out in vitro. The ability to temporally manipulate Eomes protein expression in combination with cell marking by the mCherry-reporter offers a powerful tool for dissecting Eomes-dependent functional roles in these diverse cell types in the early embryo.

## Significance

Eomesodermin (Eomes) is a T-box transcription factor essential for numerous developmental processes in the mammalian embryo. It is required in multiple extra-embryonic and embryonic cell lineages within a short window of time, which hampers efforts to understand its functional role in each lineage. Here, we describe a mouse embryonic stem cell and mouse line harboring a degron-tagged Eomes allele, which enables rapid and reversible protein degradation upon administration of a small molecule [dTAG (degradation fusion tag)]. We suggest this is a powerful tool to dissect Eomes functions at different developmental stages and cell lineages.

The T-box transcription factor (TF) Eomesodermin (Eomes) plays essential roles in multiple cell lineages of the developing embryo. Eomes is initially induced in the trophectodermal cell lineage coincident with the emergence of the blastocyst, and expression continues in the extraembryonic ectoderm-marking progenitors of the placental chorion (1–3). Eomes plays essential roles downstream of Cdx2 during expansion of the polar trophectoderm (TE). Consequently, loss-of-function mutant mouse embryos fail at implantation (2, 4, 5). Studies exploiting a conditional allele (5) in combination with various Cre-recombinase deleter strains have further revealed its essential tissue-specific roles. Transient Eomes expression in the visceral endoderm (VE) during early post-implantation development promotes the formation of the anterior VE (AVE) signaling center and orientation of the anterior–posterior axis (6). Slightly later, during the formation of the primitive streak (PS), Eomes induction downstream of Nodal/Smad signals (7) is required for nascent mesoderm cells to down-regulate E-cadherin expression and undergo epithelial to mesenchymal transition (5). Fate mapping experiments have shown that transient and dynamic Eomes expression during PS elongation sequentially delineates the progenitors of the yolk sac mesoderm, heart, definitive endoderm, and the node and midline (8).

In vitro-directed differentiation experiments exploiting Eomes-deficient embryonic stem cells (ESCs) have confirmed that transient Eomes expression has essential lineage-specifying roles. Eomes regulates the competence of the yolk sac mesoderm to give rise to the first two waves of embryonic blood formation (9). Eomes-deficient ESCs are blocked in their ability to give rise to the cardiac progenitors, in part due to the requirement for Eomes-dependent activation of the basic helix-loop-helix TFs Mesp1/2 (8). However, it has proven difficult to precisely define the temporal requirements for these activities, and the underlying molecular mechanisms by which Eomes regulates diverse developmental trajectories in the context of the embryo.

Recent experiments have exploited acute protein degradation systems via the addition of so-termed “degron” sequences to endogenously expressed genes to manipulate protein function in a wide range of settings. These systems include the auxin-inducible degron (AID) (10–12), GFP-tag (13), HaloPROTAC (14), and FKBP12^F36V^/dTAG (degradation fusion tag) (15). Recent reports demonstrated the utility of the FKBP12^F36V^dTAG system to rapidly and efficiently degrade the ubiquitously expressed elongation factor Nelfb via the proteasome pathway in cultured cells, embryos, and adult tissues (16, 17). These studies revealed unappreciated roles of Nelfb-mediated Pol II pausing in regulating pluripotency cell states in the early postimplantation embryo (17).

Here, we tested the dTAG system for its ability to rapidly and efficiently remove endogenous Eomes function in a temporally controlled fashion in the context of both differentiated ESCs and the intact embryo. We engineered an in-frame FKBP12^F36V^ tag, in combination with the self-cleaving 2A peptide followed by an mCherry fluorescent protein into the Eomes locus to generate a unique allele encoding C-terminally degron-tagged Eomes protein coexpressing the mCherry reporter. We initially analyzed homozygous Eomes^deg/deg^ ESCs in haematopoietic mesoderm differentiation assays (9). Addition of the small molecule dTAG-13 resulted in rapid and efficient Eomes degradation and concomitant loss of haemogenic potential. To evaluate the functional activities of this allele in vivo, we next generated an Eomes^deg/deg^ mouse line. Treatment of preimplantation blastocyst stage embryos with dTAG-13 in vitro results in loss of Eomes protein and failure to form outgrowths, recapitulating the null phenotype.

However, administration of either dTAG-13 or dTAG^V^-1 to pregnant females between 5.5 to 7.5 days post-coitum (dpc) failed to consistently reduce Eomes protein levels in developing postimplantation embryos. Rather, we observe highly variable levels of protein degradation, with the VE proving most sensitive to Eomes loss. Time course dTAG wash-out experiments in vitro using both embryos and ESC-derived embryoid bodies (EBs) revealed that Eomes protein levels recover rapidly within 1 to 2 h of dTAG-13 removal. Thus, the efficiency with which developmentally regulated genes can be degraded in vivo using the dTAG system is likely to be gene-specific and contingent on parameters such as steady-state transcription rates and/or levels of protein turnover. However, the ability to precisely manipulate Eomes protein function, coupled with the ability to recover acutely depleted Eomes expressing cell populations labeled by mCherry expression, provides a tractable experimental system for studying temporal functions of Eomes in both in vitro and ex vivo settings.

## Results

### Degron-Tagged Eomes Functions Normally to Support the Specification of Haematoendothelial (HE) Progenitors In Vitro.

The dTAG system (15, 18) exploits a modified FKBP12 protein, FKBP12^F36V^, as a “degron tag” to target proteins for degradation. Treatment with a small heterobifunctional molecule (such as dTAG-13 or dTAG^V^-1) mediates an interaction between the tag and an E3 ubiquitin ligase (CRBN and VHL for dTAG-13 and dTAG^V^-1, respectively), resulting in rapid ubiquitination and degradation of the tagged protein.

**Fig. 1.**
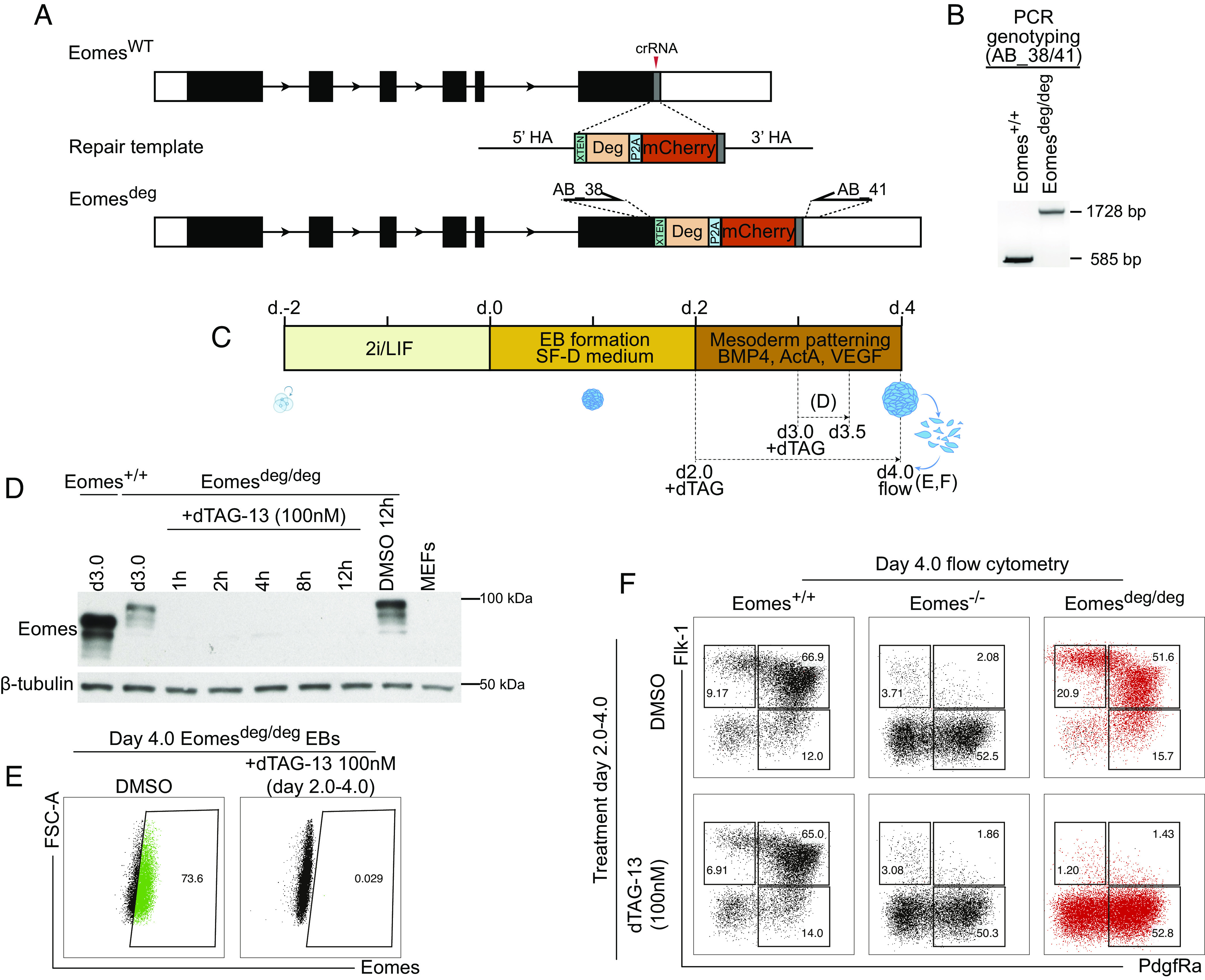
Generation of an Eomes^deg^ allele using CRISPR-Cas9 and functional analysis in vitro during HE differentiation. (*A*) Cas9-based targeting strategy for generation of the Eomes^deg^ allele. A crRNA targeting the stop codon (in grey) of the endogenous Eomes locus was used along with a ssDNA repair template containing the sequence for the degron (FKBP12^F36V^) tag, a 2A self-cleaving peptide, and an mCherry reporter. HA, homology arm; XTEN, unstructured peptide linker; P2A, self-cleaving peptide; DEG, degron tag; AB_38/41, primers used for screening of clones in panel (*B*). (*B*) PCR genotyping of wildtype and homozygously tagged ESC clones with AB_38/41 primers denoted in *A*. (*C*) Schematic of the HE EB differentiation protocol described in Harland et al. (9). Also indicated are the experimental procedures for the results presented in *D*–*F*. (*D*) Western blot analysis of day 3.0 EBs demonstrates the size shift due to the presence of the degron tag compared to Eomes^+/+^ EBs and the rapid loss of Eomes protein within 1 h of dTAG-13 treatment. Dimethylsulfoxide (DMSO) is a vehicle control and MEFs are used as a Eomes negative control. (*E*) Intracellular flow cytometry in day 4.0 Eomes^deg/deg^ EBs incubated in dTAG-13-containing medium from day 2.0 onwards demonstrates loss of Eomes protein. (*F*) Eomes^deg/deg^ EBs and parental E14-RV Eomes^+/+^ ESCs form similar cell populations during HE differentiation, as assessed by live cell flow cytometry for mesodermal/HE markers Flk1 and PdgfRa. Eomes^deg/deg^ EB cultures treated with dTAG-13 from day 2.0 onwards phenocopy Eomes^–/–^ EBs, as addition of dTAG-13 to Eomes^deg/deg^ EBs on day 2.0 results in failure to upregulate Flk1 and disrupts the formation of Flk1^+^/PdgfRa^+^ HE progenitors on day 4.0. mCherry+ cells identify the Eomes expressing cell population (red).

Here, we used Cas9-mediated targeting in combination with a single-stranded DNA (ssDNA) repair template in feeder-free E14-RV ESCs (9) to introduce the FKBP12^F36V^ degron tag at the C-terminus of the Eomes coding sequence, upstream of the stop codon. The degron sequence is followed by a self-cleaving 2A peptide and an mCherry fluorescent reporter (Fig. 1*A*). The correct structure of the knock-in allele was confirmed via PCR of the insert (Fig. 1*B*). As Eomes is not expressed in ESCs, we exploited homozygous Eomes^deg/deg^ ESCs in conjunction with a previously described EB HE differentiation protocol (Fig. 1*C*) that promotes the upregulation of Eomes expression beginning on day 2.5 (9). Starting at day 3.0, EBs were treated with dTAG-13 for various lengths of time. Western blot analysis revealed the degron-tagged Eomes protein is size-shifted due to the additional molecular weight of the tag. Addition of dTAG-13 results in complete loss of Eomes protein within 1 h of treatment (Fig. 1*D*). As assessed by intracellular flow cytometry on day 4.0 of culture, control EBs are 74% Eomes-positive, whereas in contrast, EBs treated with dTAG-13 on days 2.0 to 4.0 completely lose Eomes protein (Fig. 1*E*). Furthermore, control vehicle–treated Eomes^deg/deg^ EBs phenocopied Eomes^+/+^ EBs, showing upregulation of mesodermal marker expression (Flk1 and PdgfRa). As for Eomes-null EBs, EBs treated with dTAG-13 on days 2.0 to 4.0, fail to upregulate Flk1 and lack the ability to generate Flk1/PdgfRa double-positive haemogenic progenitors (Fig. 1*F*).

### Homozygous Eomes^deg/deg^ Blastocysts Treated with dTAG Rapidly Lose Eomes Protein.

Eomes^deg/deg^ ESCs generated using the CCE line (19) were injected into blastocysts to generate germline chimeras. These were crossed with wild-type animals to obtain heterozygous offspring that were then intercrossed to obtain homozygous Eomes^deg/deg^ animals. Pups from both heterozygous Eomes^deg/+^ x Eomes^deg/+^ and Eomes^deg/deg^ x Eomes^+/–^ intercrosses were obtained at the expected Mendelian ratios (*SI Appendix*, Fig. S1 *B* and *D*). Moreover, litter sizes from Eomes^deg/deg^ homozygous crosses were comparable to wild type (*SI Appendix*, Fig. S1*C*). Collectively, these results demonstrate that the degron-tagged Eomes protein functions normally in vivo, and this manipulation does not create a dominant-negative allele.

**Fig. 2.**
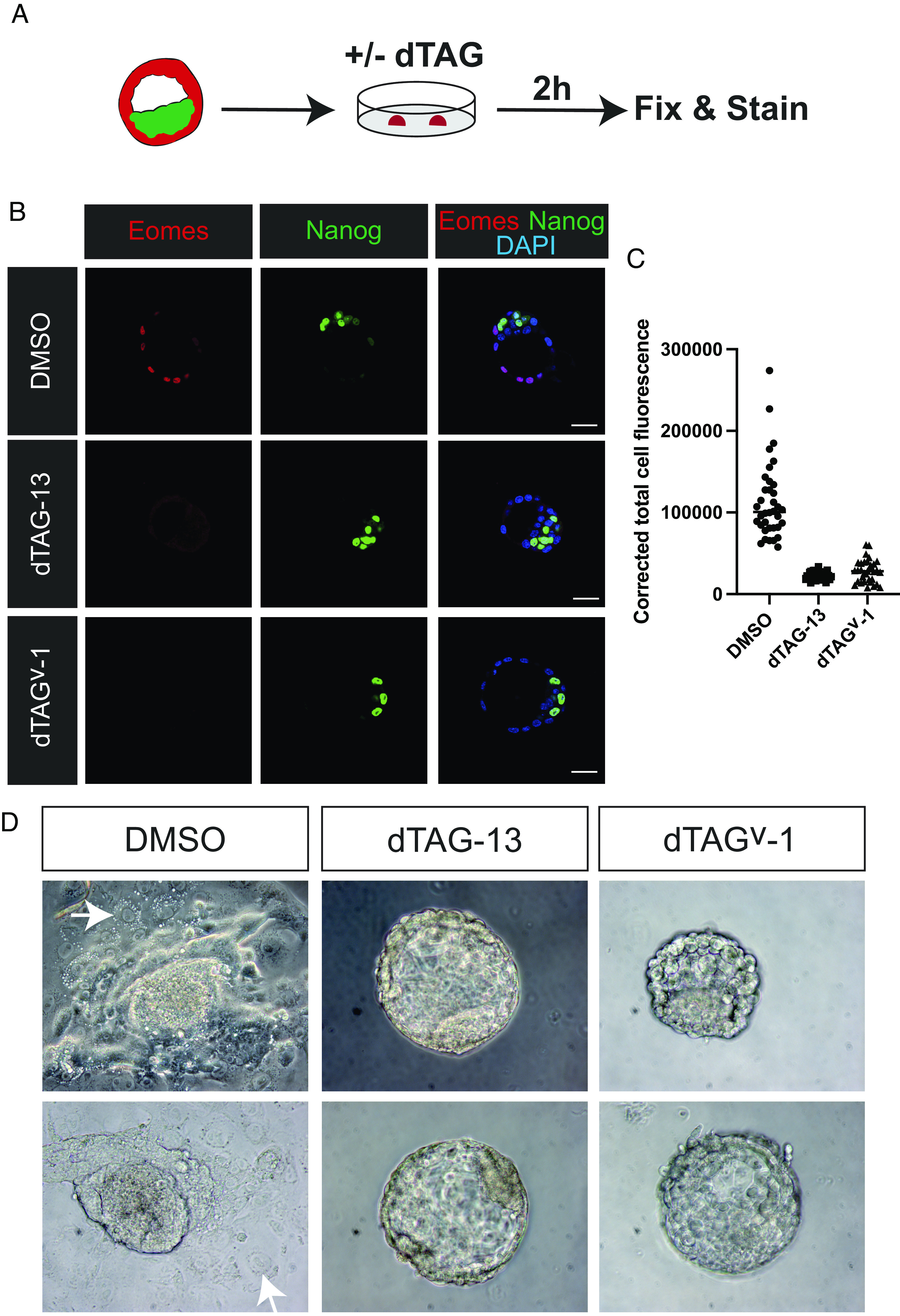
Ex vivo treatment of Eomes^deg/deg^ preimplantation embryos. (*A*) Schematic representation of the experimental protocol to test Eomes-degron degradation in blastocyst stage embryos. (*B*) Eomes and Nanog staining of DMSO, dTAG-13, or dTAG^V^-1 treated E3.5 Eomes^deg/deg^ blastocyst stage embryos. The TE of dTAG-treated Eomes^deg/deg^ embryos lacks Eomes expression. (Scale bars, 50 µm.) (*C*) Quantification of Eomes expression in Eomes^deg/deg^ blastocysts treated with DMSO (n = 5), dTAG-13 (n = 6), or dTAG^V^-1 (n = 5). Corrected total cell fluorescence is calculated as the Integrated Density – (Area of selected cell × Mean fluorescence of background readings). (*D*) Cultured Eomes^deg/deg^ homozygous blastocysts treated with DMSO, dTAG-13, or dTAG^V^-1 for 72 h. Eomes^deg/deg^ blastocysts treated with DMSO attach to the plastic substrate and form trophoblast outgrowths with typical giant cells (arrows). In contrast, Eomes^deg/deg^ blastocysts treated with dTAG-13 or dTAG^V^-1 fail to attach and outgrow phenocopying Eomes null embryos, as described previously (2, 4, 5). DMSO, n = 6; dTAG-13, n = 7; dTAG^V^-1, n = 8.

Next, we cultured Eomes^deg/deg^ homozygous E3.5 blastocysts to evaluate dTAG-dependent Eomes degradation (Fig. 2*A*). Eomes expression was readily detectable via immunofluorescence (IF) in the TE of control blastocysts (Fig. 2*B*), whereas embryos cultured for 2 h in medium containing dTAG-13 or dTAG^V^-1 entirely lack Eomes protein (Fig. 2 *B* and *C*). Moreover, culture assays carried out over 72 h demonstrate the ability of control Eomes^deg/deg^ blastocysts to generate normal outgrowths. In contrast, dTAG-13- and dTAG^V^-1-treated blastocysts phenocopy Eomes-null blastocysts (2, 4, 5) and entirely lack the ability to attach and outgrow (Fig. 2*D*).

### dTAG Treatment of Eomes^deg/deg^ Embryos In Utero Results in Variable Loss of Eomes Protein.

We next tested the impact of dTAG delivery to pregnant mice via intraperitoneal (IP) injection (16) on Eomes protein degradation. Eomes^deg/deg^ females mated to homozygous males were injected with dTAG-13 at 5.5 dpc. At this stage, Eomes is normally expressed in the TE-derived extraembryonic ectoderm (ExE) and the embryonic VE (EmVE) (1, 3). Eomes is essential during pre- and peri-implantation development. The TE domain fails to expand in null mutants and these embryos die at peri-implantation (2, 4, 5), and overall exhibit an arrested phenotype at E5.5 (*SI Appendix*, Fig. S2*A*).

However, in embryos collected and stained for Eomes 2 or 4 h after dTAG-13 injection, we found no evidence for changes in Eomes protein levels relative to control embryos (*SI Appendix*, Figs. S2*B* and S3*A*). Shortly after implantation, the embryo is known to be protected by the so-termed primary decidual zone (PDZ), an avascular region of maternal decidual cells associated with a high density of tight junctions (20). Thus, one possibility is that at this stage, the PDZ forms a barrier which renders the embryo impervious to circulating maternal dTAG-13.

**Fig. 3.**
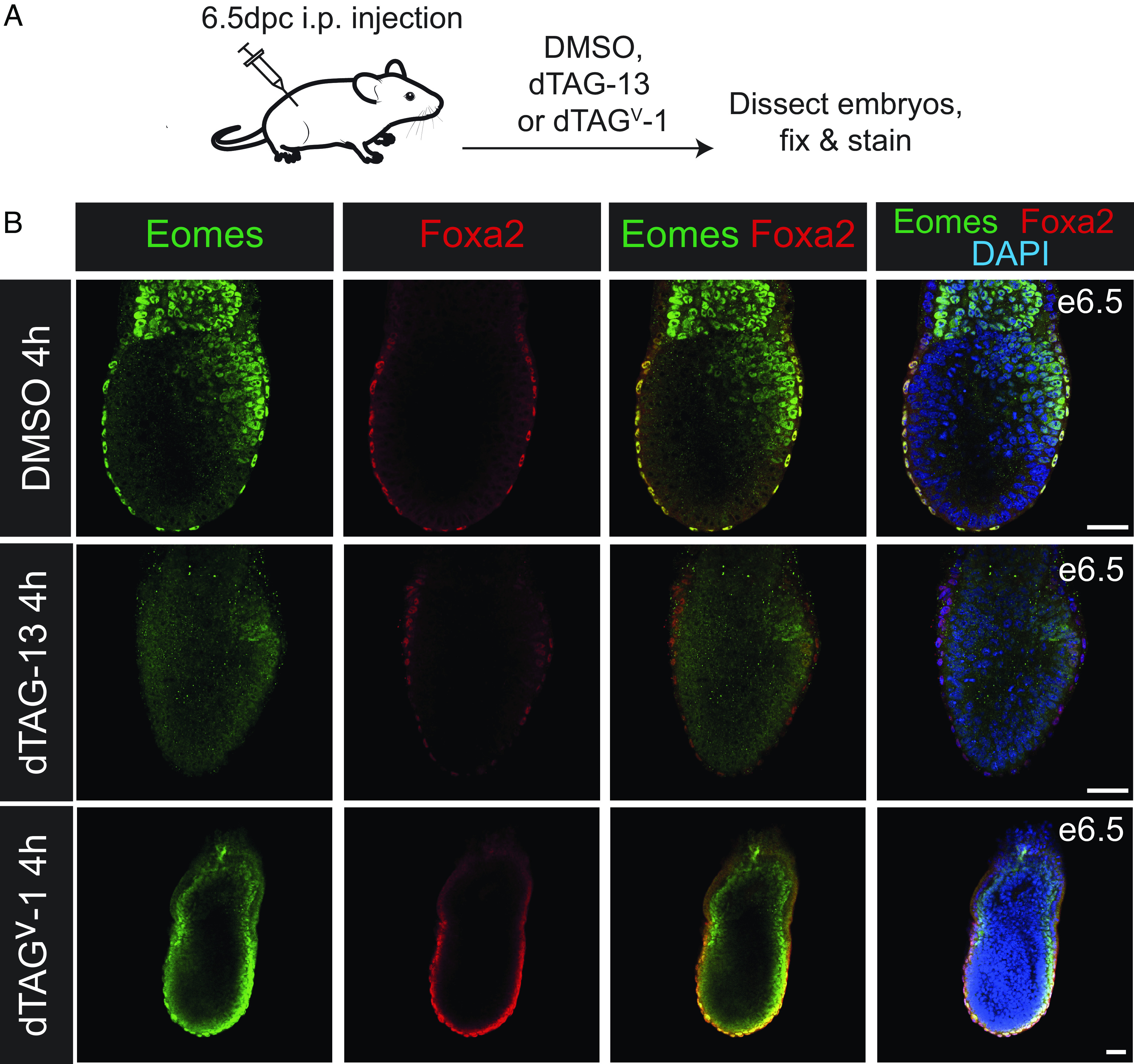
dTAG treatment of in utero embryos at 6.5dpc. (*A*) Schematic representation of the experimental protocol designed to test Eomes-degron degradation in utero at 6.5 dpc. (*B*) Eomes^deg/deg^ homozygous embryos treated in utero with DMSO, dTAG-13, or dTAG^V^-1 for 4 h were dissected, fixed, and stained for Eomes (green) and Foxa2 (red). Nuclei are counterstained with DAPI (blue). Foxa2 staining identifies the VE and the definitive endoderm progenitors in the PS. Eomes staining shows a heterogeneous pattern of degradation of the tagged Eomes-degron protein. Selected images illustrate the range of Eomes degradation observed. Eomes positive cells are detectable in the PS on the proximal posterior side of the dTAG-13 treated embryo. The dTAG^V^ -1 treated embryo shown retains Eomes expression in the ExE, EmVE, and PS. (Scale bars, 50 µm.)

It was previously reported that injection of dTAG-13 into pregnant females at 6.5 dpc results in efficient degradation of Nelfb in the embryo (16). At this stage of development (early streak), Eomes is expressed in three distinct domains: namely, the ExE, the EmVE, and the emerging PS (1, 3). Pregnant females were injected at 6.5 dpc and embryos collected 2, 3, or 4 h later were assessed for Eomes expression via IF (Fig. 3). A few exceptional embryos exhibited a complete loss of Eomes protein. However, the majority of embryos showed only a partial loss of Eomes and a few embryos retained normal Eomes expression (*SI Appendix*, Fig. S3*B*). This variability was observed within embryos from the same litter. Thus, the efficacy of individual injections cannot account for the underlying cause of this variability. Similarly, we also see a variability in degradation in embryos where the allelic dosage has been reduced by crossing the Eomes^deg^ allele over a GFP-null allele (3) (Eomes^deg/GFP-null^) (*SI Appendix*, Fig. S4).

Interestingly, the majority of embryos across all litters exhibited complete loss of Eomes protein from the VE. Fewer exhibited decreased Eomes expression in the ExE, and also in the PS (*SI Appendix*, Fig. S3*B*). We suggest that these results probably reflect differing transcriptional and/or translational dynamics of Eomes protein in these distinct tissues.

Because E6.5 embryos treated with dTAG-13 exhibited variable loss of Eomes next we decided to test the efficacy of the dTAG^V^-1 small molecule, reported to have increased in vivo pharmacokinetics compared to dTAG-13 (18). Embryos recovered from dTAG^V^-1-treated females 2 h postinjection again exhibited variable Eomes protein depletion (*SI Appendix*, Fig. S3*B*). Moreover, extending the time period between injection and embryo collection to 4 h failed to improve Eomes depletion. Rather, the shorter 2-h timepoint resulted in comparatively higher levels of Eomes protein degradation (*SI Appendix*, Fig. S3*B*). This partial recovery of Eomes protein levels during the longer posttreatment periods, potentially reflects the clearance of the small molecule from the maternal circulation.

**Fig. 4.**
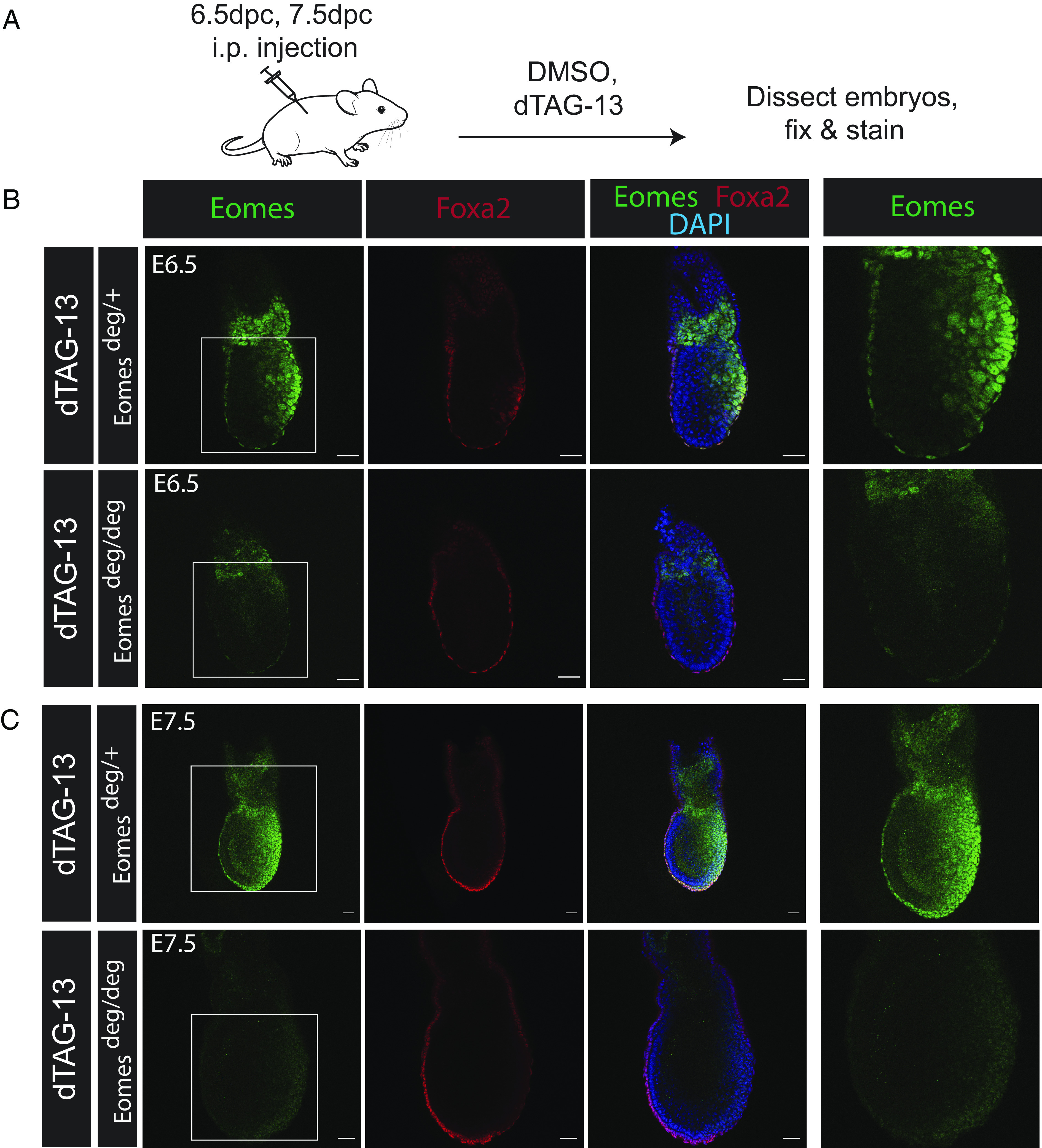
Eomes expression is differentially reduced in a spatial pattern in Eomes^deg/deg^ embryos after dTAG-13 injection at 6.5 dpc and 7.5 dpc. (*A*) Schematic representation of the experimental protocol designed to test Eomes -degron degradation in utero at 6.5 dpc and 7.5 dpc. (*B* and *C*) IF staining of E6.5 (*B*) and E7.5 (*C*) Eomes^deg/+^ and Eomes^deg/deg^ embryos for Eomes (green) and Foxa2 (red) recovered 2 h after pregnant females were injected IP with dTAG-13, as indicated in schematic diagram. White boxes indicate the magnified areas of Eomes expression displayed in the *Right* panels. Images representative of 12 embryos (E6.5) and 7 embryos (E7.5). Foxa2 (red) staining highlights VE and definitive endoderm cells. Nuclei were stained with DAPI (blue). (Scale bars, 50 μm.)

Next, we directly compared Eomes protein degradation in Eomes^deg/deg^ embryos with control nondegrading Eomes^deg/+^ littermates by crossing Eomes^deg/deg^ females with Eomes^deg/+^ males (Fig. 4*A*). Pregnant females were injected with dTAG-13 on E6.5 (Fig. 4*B*) or E7.5 (Fig. 4*C*) and embryos retrieved 2 h later were stained via IF. Consistent with results above in E6.5 Eomes^deg/deg^ embryos, the VE nearly always fully lost Eomes protein, while the ExE and PS mostly exhibited partial loss. In contrast, E7.5 Eomes^deg/deg^ embryos exhibited a more uniform loss of Eomes protein across both embryonic and extraembryonic tissues (Fig. 4*C*). Injections of dTAG^V^-1 (*SI Appendix*, Fig. S5*A*) gave a similar result, with E6.5 Eomes^deg/deg^ embryos exhibiting Eomes protein loss predominantly in the VE and the ExE and PS commonly showing partial or no loss (*SI Appendix*, Fig. S5*B*). E7.5 embryos treated with dTAG^V^-1 showed a more uniform but partial loss of Eomes, while some retained wild-type levels of expression (*SI Appendix*, Fig. S5*C*), confirming that dTAG^V^-1 treatment does not enhance protein depletion relative to dTAG-13.

### Ex Vivo dTAG Treatment of Postimplantation Embryos Fully Depletes Eomes Protein.

**Fig. 5.**
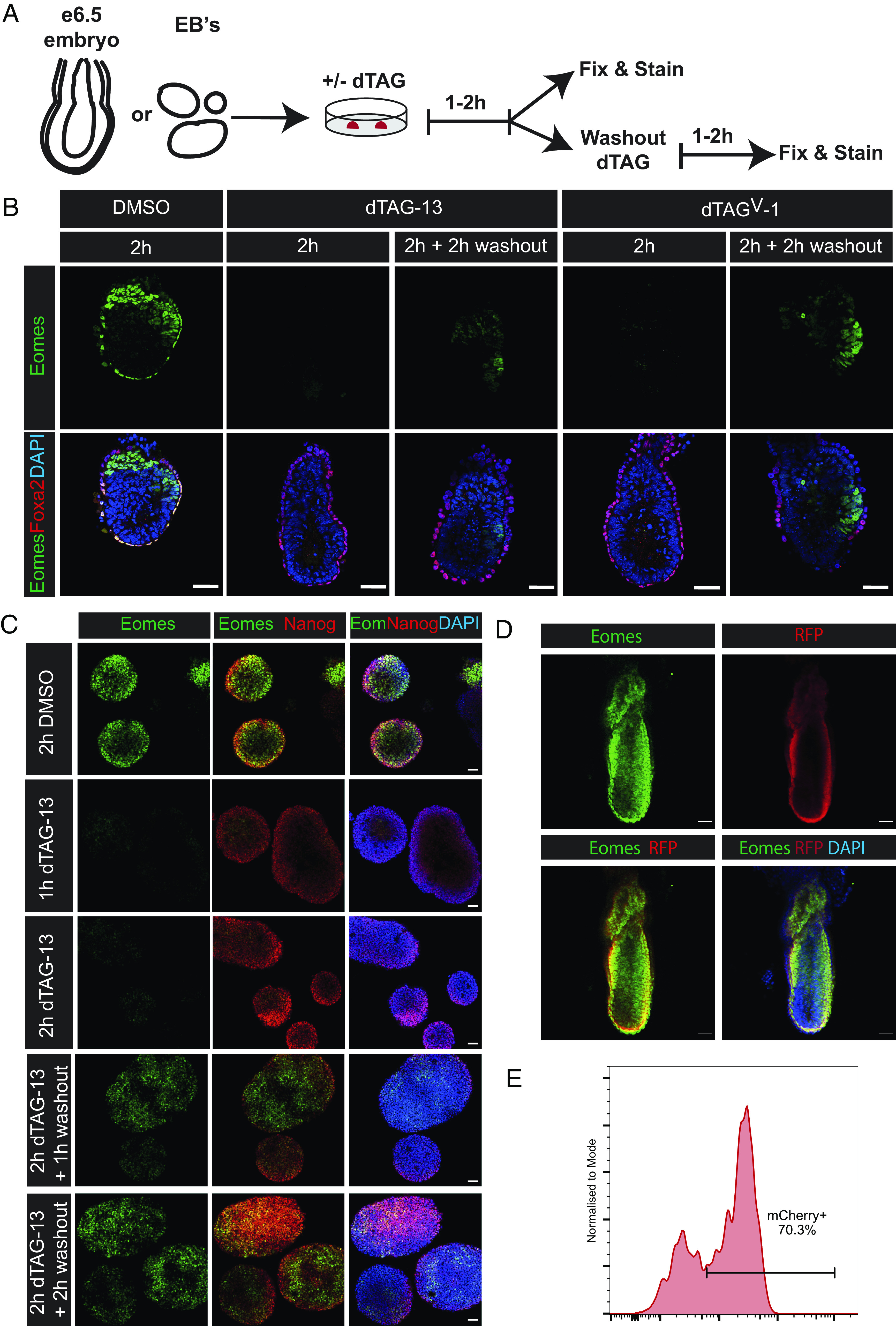
Ex vivo treatment of Eomes^deg/deg^ embryos and EBs to assess the time-course of Eomes recovery after dTAG-13 or dTAG^V^-1 washout and validation of the mCherry reporter. (*A*) Schematic representation of experimental protocol for dTAG small-molecule treatment and washout. (*B*) Eomes (green) and Foxa2 (red) staining of DMSO, dTAG-13, or dTAG^V^-1 treated E6.5 ex vivo cultured embryos fixed after 2 h or subsequently washed out and cultured for a further 2 h. Eomes expression recovers in the proximal posterior epiblast/PS region 2 h post washout. Nuclei are stained with DAPI (blue). (Scale bars, 50 μm.) (*C*) Eomes (green) and Nanog (red) staining of day 3.0 HE EBs cultured under the indicated conditions to assess the kinetics of Eomes recovery during in vitro differentiation conditions. Eomes staining is undetectable in EBs treated for 1 h and 2 h with dTAG-13. Eomes nuclear staining becomes evident 1 h post-washout onwards. Nanog staining identifies pluripotent cells. Nuclei are stained with DAPI (blue). (Scale bars, 50 μm.) (*D*) RFP (red) and Eomes (green) staining of E7.5 Eomes^deg/+^ embryos. RFP staining detects the mCherry reporter expression. Nuclei are stained with DAPI (blue). (Scale bars, 50 μm.) (*E*) Flow cytometric analysis of mCherry reporter expression in dissociated E7.5 Eomes^deg/deg^ embryos. Roughly 70% of these cells express the mCherry reporter.

To test whether the inability to reliably degrade Eomes protein in utero reflects transient exposure to dTAG from the maternal circulation, we next isolated E6.5 embryos and cultured them in dTAG-containing medium (Fig. 5*A*). In keeping with this suggestion, after a 2-h incubation, ex vivo Eomes protein was completely undetectable in embryos treated either with dTAG-13 or dTAG^V^-1 (Fig. 5*B*).

In the case of the broadly expressed gene Nelfb, protein levels in embryos treated in utero via a single administration of dTAG recover slowly over the following 24 h (16). To examine the kinetics of the reversibility of Eomes protein expression we performed wash-out experiments ex vivo. Following a 2-h incubation in dTAG-containing medium to ensure complete removal of Eomes protein, embryos were washed to remove residual dTAG, cultured for additional periods of time, and assessed for Eomes protein via IF. While undetectable 1 h later (*SI Appendix*, Fig. S6*B*), we observed the reappearance of immuno-reactive Eomes protein after only 2 h. Recovery was observed primarily in the posterior epiblast, and to a lesser degree in the ExE, while Eomes remained undetectable in the VE (Fig. 5*B*).

To further investigate the kinetics of Eomes protein recovery, we used the HE in vitro differentiation protocol in which Eomes transcription is robustly upregulated in EBs in response to the addition of exogenous growth factors (Fig. 1*C*). In day 3.0 EBs incubated in dTAG, we observed similarly rapid Eomes protein recovery after washout (Fig. 5*C*). Eomes was totally undetectable within 1 h of dTAG-13 addition, while Eomes-positive cells were observed as early as 1 h after dTAG-13 washout, with substantial recovery to almost wild-type levels observed after only 2 h (Fig. 5*C*). Thus, newly transcribed and translated Eomes mRNA rapidly replenishes the nuclear Eomes protein pool.

Finally, to assess the utility of the mCherry reporter in vivo, we carried out IF costaining for Eomes and the mCherry reporter. As expected, Eomes and mCherry are co-expressed and overlap significantly at E7.5 (Fig. 5*D*). We also performed live cell flow cytometry of cells from dissociated E7.5 Eomes^deg/deg^ embryos (Fig. 5*E*). This showed that approximately 70% of cells are mCherry-positive and clearly distinguishable from the remaining mCherry-negative cells, thus allowing Eomes-expressing cells to be selectively retrieved from the developing embryo.

## Discussion

The generation of degron-tagged alleles, a powerful approach for analyzing tissue-specific gene function, offers several distinct advantages over traditional genetic methods. In contrast to the use of tissue-specific or inducible Cre transgenes or alleles to conditionally delete genes of interest, this methodology offers the opportunity to immediately and acutely manipulate protein expression in vivo in a temporally controlled manner. Moreover, this approach bypasses the requirement for generating and maintaining mouse strains harboring multiple alleles and obviates the necessity for genotyping the resulting tissues or embryos. Furthermore, the reversible nature of the dTAG system by simple washout of the small molecule allows the downstream consequences of protein perturbations to be assessed in developmental settings.

Here, we describe the generation of ESC lines and viable mice carrying a degron-tagged allele of the T-box TF Eomes. As expected, the addition of either dTAG-13 or dTAG^V^-1 to cultured cells, or pre-implantation stage embryos, results in rapid and efficient loss of Eomes function. Similarly, Eomes^deg/deg^ postimplantation embryos treated ex vivo with dTAG-13 or dTAG^V^-1 lose Eomes protein within 2 h in all sites of Eomes expression. dTAG-treated Eomes^deg/deg^ EBs lack the ability to undergo Eomes-dependent specification of haematopoietic progenitors in vitro, and dTAG-treated cultured Eomes^deg/deg^ blastocysts recapitulate the null phenotype.

However, in contrast to recent reports, we find here that in utero treatment by IP injection of either dTAG-13 or dTAG^V^-1 (16) fails to efficiently promote Eomes degradation. E5.5 embryos showed normal levels of Eomes protein 2 or 4 h after small-molecule administration to pregnant females. At this very early stage, one possibility is that the largely avascular PDZ surrounding the newly implanted embryo forms an impervious barrier preventing diffusion of dTAG from the maternal circulation. Consistent with this idea, it has been suggested that the presence of the “testes blood barrier” accounts for inefficient AID-mediated degradation of Ncaph in adult sperm in vivo (21). It will be important to test whether other tagged proteins may prove similarly recalcitrant to AID- or dTAG-mediated degradation at this early stage of in utero development. By contrast, at later stages, namely E6.5 and E7.5, we observed dTAG-dependent loss of immunoreactive Eomes protein. However, this outcome proved to be highly variable even within individual litters. Small-molecule bioavailability is unlikely to be the explanation for this finding since neither dTAG-13 nor dTAG^V^-1 (which target different components of the proteasome pathway) promotes efficient and reproducible protein elimination.

Interestingly, we found that Eomes expression in the VE tissue was especially sensitive to dTAG degradation. Eomes is only briefly transcribed in this lineage where it acts at E5.5-6.0 to induce the AVE (6). In contrast, Eomes is very robustly expressed in the ExE and epiblast between E6.5 to E7.5 as demonstrated by in situ mRNA hybridization and GFP-reporter expression (1, 3). Consequently, transient exposure to high levels of circulating maternal dTAG may be insufficient to promote Eomes degradation in these tissues.

In contrast to the present findings, recent experiments targeting the Nelfb protein, a member of the RNAPII complex widely expressed both in the developing embryo and adult mouse, demonstrated complete degradation of Nelfb protein in homozygous degron-tagged Nelfb embryos following a single IP injection of pregnant mice at 6.5, 8.5, and 10.5 dpc. Protein recovery postinjection gradually returned to approximately 70% that of the original levels only 24 h later (16). Similarly, in a system employing an auxin-inducible degron of Ncaph, a subunit of the structural maintenance of chromosome complex condensin (21), single administration of the auxin analogue IAA resulted in almost complete (>90%) loss of AID-tagged Ncaph in CD8+ thymocytes within 2 h. Expression gradually recovered to near-normal levels within 72 h. Our ex vivo washout experiments in both embryos and EBs demonstrate that Eomes protein recovery is very rapid and readily detected within 1 to 2 h following dTAG removal. Thus, the efficacy of dTAG-mediated protein degradation is likely to be highly dependent on constitutive steady-state expression levels of the target protein and its in vivo turnover rate.

The key lineage-determining TF Eomes is dynamically expressed during early postimplantation development. Fate mapping studies have shown that its expression within the epiblast marks multiple lineages including the progenitors of the haemogenic yolk sac mesoderm, cardiogenic mesoderm, the definitive endoderm, and the anterior mesendoderm/axial mesoderm (5, 8, 9, 22). These functionally distinct progenitor populations become specified sequentially during gastrulation within a narrow 24 to 36 h timeframe. Targeted deletion of Eomes in the early epiblast using the Sox2.Cre strain arrests development at the onset of gastrulation (5). This early block in nascent mesoderm delamination leaves open the question as to how Eomes acts to regulate divergent cell fate allocation during the subsequent 36-h period of development.

The Eomes^deg/deg^ mouse strain provides a versatile tool that offers the opportunity to acutely deplete and/or restore Eomes protein at distinctive developmental timepoints ex vivo or during directed in vitro differentiation. Coupled with the inclusion of the mCherry reporter, this allele readily allows the recovery of Eomes wildtype vs. depleted cell populations. Single-cell experiments together with multiomic approaches (23) should hopefully allow the identification of Eomes-dependent gene regulatory networks that regulate haemogenic mesoderm, cardiac, and definitive endoderm fates. The establishment of culture conditions that permit the development of mouse embryos from early gastrulation through to early somite stages (24, 25) should allow the morphological consequences of Eomes depletion at different timepoints to be observed in real time. Additional work will be needed to test whether this degron-based approach may prove useful for understanding underlying mechanisms that allow other TFs to regulate cell lineage fate choices during the emergence of the embryonic body plan.

## Materials and Methods

### Generation of the *Eomes-degron-P2A-mCherry* Repair Template for Cas9 Targeting.

An IDT Megamer® was designed to contain the sequence for the Eomes-degron-P2A-mCherry KI allele flanked by homology sequences for the 5′ (the 3′ end of the Eomes exon 6 coding sequence (CDS)) and 3′ (3′ untranslated region (UTR)) homology arms. This was amplified with primers containing restriction digest sites EcoRV and Nb.BsmI on the 5′ end of the repair template (5′-EcoRV- Nb.BsmI-3′) and NotI on the 3′ end of the repair template. The PCR product and a pBlueScript KS- plasmid were then digested with EcoRV and NotI (New England Biolabs), and the resulting fragment was ligated into the plasmid with NEBuilder HiFi DNA Assembly Master Mix (New England Biolabs) and amplified in NEB Stable cells (New England Biolabs) following the manufacturer’s instructions. The plasmid was digested with Nb.BsmI and NotI, denatured using Denaturing Gel-loading Buffer (Biodynamics Laboratory), and run out on an agarose gel, from which the single-stranded fragment was excised and extracted using the Zymoclean™ Gel RNA recovery kit (Zymo Research). The single-stranded oligonucleotide (ssODN) was freeze-dried, resuspended in IDT duplex buffer, Nanodropped, and used as a repair template at 0.5 pmol.

### Cas9-Mediated ESC Modification.

Custom synthetic CRISPR RNA (crRNA) and ssODN (Integrated DNA Technologies, USA) were resuspended in IDT duplex buffer. ESC cultures were changed into antibiotic-free media 6 h prior to electroporation. Cas9 ribonucleoprotein (RNP) complexes were assembled immediately prior to electroporation following the manufacturer’s protocol (Alt-R CRISPR-Cas9 System: Delivery of RNP complexes into Jurkat T cells using the Neon® Transfection System). Briefly, 4.4 μL of 100 μM tracrRNA, 4.4 μL of 100 μM crRNA, and 1.2 μL of IDT Duplex Buffer were mixed, heated at 95 °C for 5 min using a thermocycler, and then cooled to room temperature. The RNP complex was formed by diluting Alt-R® S.p. HiFi Cas9 Nuclease V3 in Buffer R to 36 μM and mixing 1:1 with the crRNA:tracrRNA duplex. Then, 2 × 10^5^ ESCs were resuspended in 10 μL buffer R and electroporated using the Neon transfection system (V = 1,600V, pulse width = 10 ms, number of pulses = 3) with ssODN (50 pmol) and RNP. Low-density plating was performed after 2 to 3 d. Colonies were picked after 7 to 10 d and screened by PCR to identify putative homozygous clones. These were further verified by PCR amplification of the full-length insert followed by Sanger sequencing.

### ESC Maintenance and Differentiation.

Feeder-independent ESC lines E14-RV (9) and Eomes^–/–^ (5) were maintained in serum/LIF conditions as previously described (8). Cells were dissociated in 0.25% Trypsin (Gibco) and passaged every 2 d. Cells were plated at a density of 0.6 × 10^6^/mL, and 48 h prior to differentiation, the medium was replaced with serum-free ESC media containing 50% Neurobasal medium (Gibco), 50% DMEM (Dulbecco's Modified Eagle Medium)/F12 (Gibco), and supplemented with 0.5× N2 (Gibco), 0.5× B27 (Gibco), 1% Pen/Strep (Gibco), 1% L-glutamine (Gibco), 0.05% Bovine Serum Albumin/BSA (Gibco), 1 μM PD-0325091 (University of Dundee, MRC-PPU unit), 3 μM CHIR99021 (University of Dundee, MRC-PPU unit), and 1,000 U/mL Leukemia inhibitory factor/LIF (Millipore). After 2 d of further culture, cells were washed with PBS (phosphate-buffered saline) (Gibco), dissociated with TrypLE (Gibco), seeded at a density of 5 × 10^4^/mL in SF-D medium (26) on 6 cm non-TC-treated dishes (Thermo Fisher), and cultured on an orbital shaker at 70 rpm for 24 h in the absence of growth factors to initiate EB formation. To induce the formation of HE progenitors, d2.0 EBs were expanded into 10-cm dishes (Thermo Fisher) coated in 5% PolyHEMA (Sigma), in SF-D medium supplemented with 5 ng/mL recombinant human (rh) VEGF (R&D Systems), 10 ng/mL rhBMP4 (R&D Systems), and 5 ng/mL Activin A (R&D Systems). To assess Eomes protein degradation in EBs, the small-molecule dTAG-13 (Bio-Techne) was resuspended in DMSO per the manufacturer’s instructions and then diluted to a final concentration of 100 nM in a culture medium. DMSO alone was used as a vehicle control. Feeder-dependent CCE ESCs were maintained as described (19), electroporated, and low-density plated, and the resulting colonies were screened as described above.

### Western Blot.

EBs were lysed in RIPA buffer (50 mM Tris pH8.0, 150 mM NaCl, 1% Igepal, 0.5% sodium deoxycholate, 0.1% Sodium dodecyl sulfate(SDS)). Protein was quantified by Bradford assay with the DC™ Protein Assay Kit I (Bio-Rad) on a Jenway Genova DNA Life Science Analyser. Samples were denatured at 98 °C for 10 min in Laemmli sample buffer (BioRad) with 10% β-mercaptoethanol, run at 90 V on a Mini-Protean® PAGE gel (BioRad), and transferred onto PVDF membrane for 75 min at 90 V. The membrane was rinsed with distilled H_2_O, washed in Tris-buffered saline-Tween20 (TBST) (0.1%) for 10 min, blocked with EveryBlot blocking buffer (BioRad) for 10 min at RT (room temperature), and incubated in primary antibody on a shaking platform at 4 °C overnight. The membrane was washed with TBST, incubated in secondary antibody in blocking buffer, washed in TBST and ECL prime (Sigma-Aldrich) was added per the manufacturer’s instructions, and exposed to X-ray film. When necessary, the membrane was stripped with stripping buffer (2.9 g glycine, 20 mL SDS in 2 L H_2_O, pH 2.2), blocked, washed, and exposed to antibody as described above.

### Flow Cytometry.

For live cell flow cytometry, EBs were washed with PBS, dissociated in TrypLE and neutralised in FACS buffer (PBS supplemented with 1% Pen/Strep, 2% Fetal Calf Serum (FCS)). Cells were resuspended in FACS buffer containing fluorophore-conjugated PdgfRa and Flk1 antibodies and stained on ice for 30 min. Cells were washed and resuspended in FACS buffer containing 1:5,000 DAPI (BD Biosciences) for 15 min on ice, then washed and resuspended in FACS buffer prior to analysis.

For intracellular flow cytometry, cells were dissociated and stained with a viability dye (ThermoFisher) and fixed using Fixation/Permeabilization Concentrate and Diluent (eBioscience) for 40 min on ice, protected from light. Cells were washed in FACS buffer and permeabilized using Permeabilization Buffer (eBioscience). Cells were incubated with Eomes antibody at room temperature for 30 min, protected from light, washed, and resuspended in FACS buffer.

E7.5 Eomes^deg/deg^ embryos were harvested in M2 media (Sigma), and the Reichert’s membrane and ectoplacental cone were removed. Embryos were washed in Mg/Ca-free PBS/2% FCS and dissociated in 0.25% Trypsin-EDTA for 10 min at 37 °C, by gentle trituration. The resultant cell suspension was neutralized in M2/FCS solution, pelleted at 1,000 rpm for 3 min, washed twice in Mg/Ca+ PBS, and resuspended in FACS buffer. Cells were analysed on a BD Fortessa X20. Data analysis was carried out on FlowJo™ v10.8.1 Software (BD Life Sciences). Antibodies used are listed in *SI Appendix*, Table S1.

### Animals.

Chimeric mice were generated by injecting two independent CCE-derived homozygous Eomes^deg/deg^ clones into C57BL/6J blastocysts. The resulting chimeric males were mated to sexually mature C57BL/6J females. Agouti offspring were genotyped (*SI Appendix*, Table S2, Primers) to detect the transmission of Eomes^deg^ allele. Eomes^deg/+^ mice were intercrossed to generate Eomes^deg/deg^ offspring, which were then used to establish an Eomes^deg/deg^ colony. Sexually mature females (at least 6 wk of age) and males (at least 7 wk of age) of the indicated genotype were intercrossed. Eomes^+/–^ and Eomes^GFP^ mice were genotyped as described (3, 5). Day of vaginal plug was defined as 0.5 days post coitum (dpc). All animal experiments were authorized by the Oxford Local Animal Welfare and Ethical Committee under Project License PF2B5F15 and performed in accordance with Home Office (UK) guidelines.

### Eomes Degradation in Embryos In Vitro and In Vivo.

dTAG-13 or dTAG^V^-1 was dissolved in DMSO at 12.5 mM and further diluted in DMEM (Gibco) at 500 nM. E3.5 or E6.5 Eomes^deg/deg^ embryos were harvested and incubated in DMEM or DMEM/10% FCS, respectively, with DMSO or dTAG-13/dTAG^V^-1 in a humidified 37 °C incubator with 5% CO_2_. After 2 h, the embryos were washed, fixed, and immunostained for Eomes and Nanog or Foxa2 (*SI Appendix*, Table S1). For the washout experiments, embryos were washed in DMEM/10% FCS at least 5 times and were further incubated for 2 h prior to immunostaining.

To induce Eomes degradation in E5.5, E6.5 or E7.5 embryos, a single IP injection of dTAG-13 or dTAG^V^-1 (Bio-Techne; 0.7 mg/20 g body weight) was administered. dTAG-13 or dTAG^V^-1 was reconstituted in DMSO (Sigma) at 1 mg/25 μL and then diluted in 10% Kolliphor EL (Sigma-Aldrich) in PBS. Embryos were harvested 2 to 4 h post injection. Individual yolk sac tissue was genotyped (*SI Appendix*, Table S2) by PCR to identify Eomes^deg/+^ and Eomes^deg/deg^ embryos.

### Immunofluorescence.

EBs, blastocysts, and mouse embryos (E5.5, E6.5, and E7.5) were harvested and fixed in 4% paraformaldehyde at RT for 15, 30, and 45 min, respectively. After three washes in PBS containing 0.1% Triton X-100 (PBS-T), samples were permeabilized in PBS containing 0.5% Triton X-100 at RT for 20 min, washed in PBS-T, blocked in 5% donkey serum and 0.2% BSA in PBS-T at RT for 1 h. Samples were incubated overnight with primary antibodies in blocking solution at 4 °C. After washing in PBS-T, samples were incubated with fluorophore-conjugated secondary antibodies in blocking solution for 2 h at RT, washed in PBS-T, and mounted in Vectashield with DAPI. Samples were imaged on an Olympus Fluoview FV1000 microscope and image data were processed using ImageJ software. Antibodies are listed in *SI Appendix*, Table S1.

### Statistics.

Embryos were imaged and/or stained and genotyped retrospectively to exclude any bias. The sample size (*n*) for each experiment is described in the corresponding figure legend, and all experiments were conducted with at least three biological replicates. All statistical analyses were performed using GraphPad Prism (8.2.1).

## Supplementary Material

Appendix 01 (PDF)

## Data Availability

All study data are included in the article and/or *SI Appendix*.
