# Supplementary material for: A degron-based approach to manipulate Eomes functions in the context of the developing mouse embryo
Source: Proc Natl Acad Sci U S A. 2023 Oct 23;120(44):e2311946120. doi: 10.1073/pnas.2311946120 (PMC10622880; doi:10.1073/pnas.2311946120)
Supplement: Supplementary file 1 — Appendix 01 (PDF) [file pnas.2311946120.sapp.pdf]

PNAS

Supporting Information for

A degron-based approach to manipulate Eomes functions in the context of the developing mouse embryo

Alexandra M. Bisia[1,2], Ita Costello[1,2], Maria-Eleni Xypolita[1,2], Luke T.G. Harland[1], Philipp J. Kurbel[1], Elizabeth K. Bikoff[1] and Elizabeth J. Robertson[1,3]

1. Sir William Dunn School of Pathology
University of Oxford,
South Parks Road, Oxford OX1 3RE, UK

2. A.M.B, I.C & M-E. X contributed equally to this work

3. To whom correspondence should be addressed
(elizabeth.robertson@path.ox.ac.uk)

This PDF file includes:

Figures S1 to S6
Tables S1 to S3

A

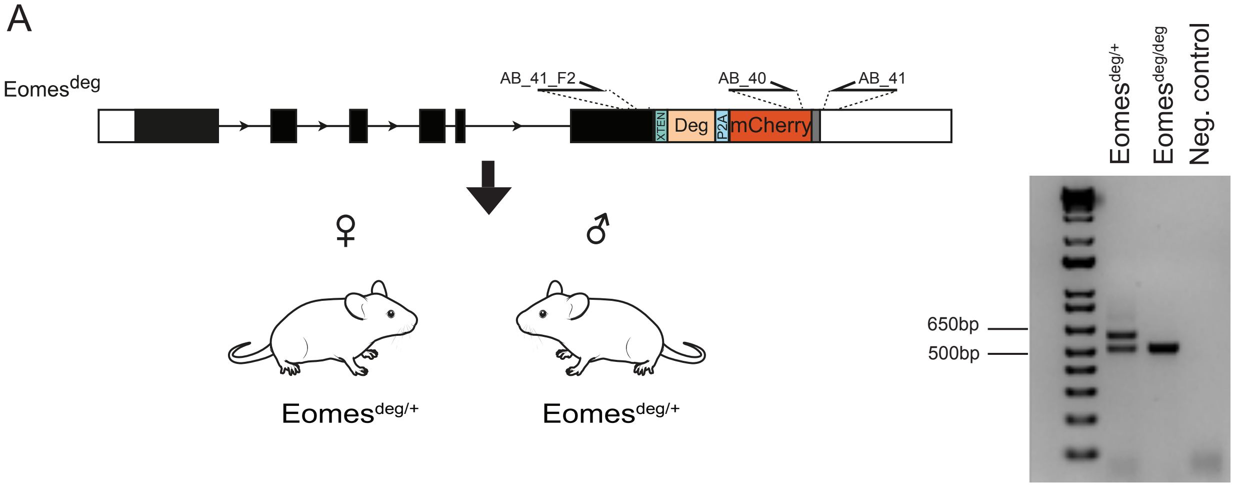

B

Genotype	No.
Eomes$^{+/+}$	24 (23%)
Eomes$^{deg/+}$	55 (52%)
Eomes$^{deg/deg}$	27 (25%)
Total	106

C

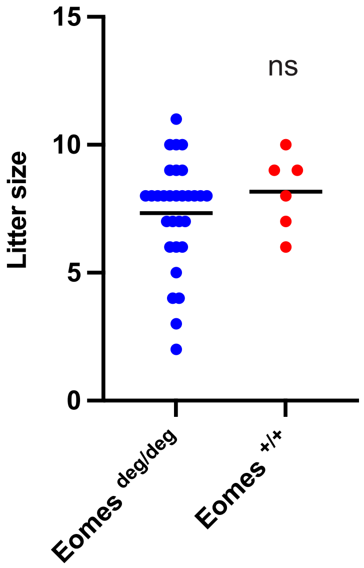

D

Genotype	No.
Eomes$^{deg/+}$	22 (48%)
Eomes$^{deg/-}$	24 (52%)
Total	46

Supplemental Figure S1: Generation of the Eomes^{deg} mouse line.

(A) Schematic of Eomes^{deg} allele. Arrows indicate location of primers used for genotyping. PCR genotyping of offspring obtained from Eomes$^{deg/+}$ intercrosses, wildtype band size is 607bp and Eomes^{deg} band size is 515bp. (B) Observed genotypes in mice derived from Eomes$^{deg/+}$ intercrosses. (C) Numbers of offspring from Eomes$^{deg/deg}$ crosses compared to wild type controls. Individual litters are shown as coloured dots (WT: red, Eomes$^{deg/deg}$: blue)**.** Statistical analysis was performed using two-tailed unpaired Student's *t*-test, p-value=0.3641, ns: not significant. (D) Genotypes of pups derived from Eomes$^{deg/deg}$ and Eomes$^{+/-}$ intercrosses.

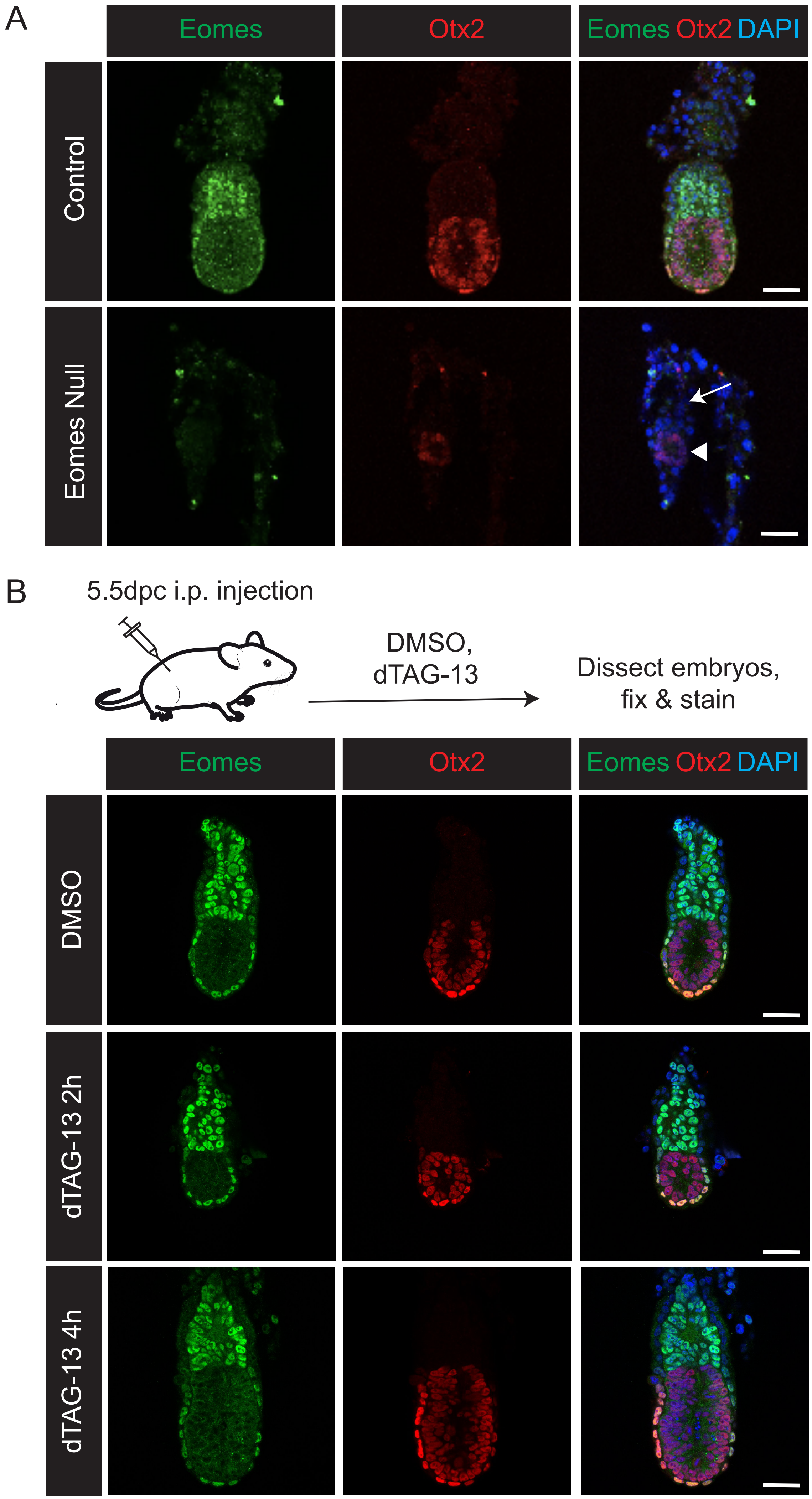
A
Eomes
Otx2
Eomes Otx2 DAPI
Control
Eomes Null
B
5.5dpc i.p. injection
DMSO,
dTAG-13
Dissect embryos,
fix & stain
Eomes
Otx2
Eomes Otx2 DAPI
DMSO
dTAG-13 2h
dTAG-13 4h

Supplemental Figure S2: dTAG-13 treatment of $Eomes^{deg/deg}$ embryos *in utero* at 5.5 dpc.

(A) Eomes (green) and Otx2 (red) staining of E5.5 control and Eomes null embryos generated from $Eomes^{+/-}$ intercrosses. Eomes is expressed in extraembryonic ectoderm (ExE) and the embryonic visceral endoderm (EmVE) in control embryos, while Otx2 is expressed in the epiblast (Epi) and overlying EmVE. Arrow indicates ExE region that fails to grow and expand in $Eomes^{-/-}$ embryos. Arrowhead indicates reduced Otx2-positive epiblast which fails to proliferate due to the absence of a functional ExE. $Eomes^{-/-}$ embryos also lack Otx2 expression in the VE.
(B) $Eomes^{deg/deg}$ embryos treated *in utero* with DMSO or dTAG-13 for 2 or 4 hours and fixed and stained for Eomes and Otx2. Eomes expression is still evident in both the ExE and EmVE in treated embryos. Otx2 expression is similarly unperturbed in dTAG-13 treated embryos. Scale bars, 50μm.

A

E5.5 embryos

dTAG	Duration	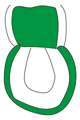	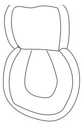
dTAG-13	2hr	100%	
dTAG-13	4hr	100%	
dTAG-13	4hr	100%	
dTAG-13	4hr	100%	

B

E6.5 embryos

dTAG	Duration	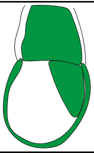	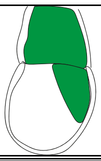	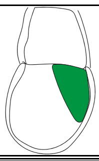	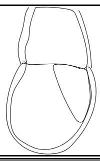
dTAG-13	2hr				100%
dTAG-13	2hr				100%
dTAG-13	2hr		67%		33%
$dTAG^{V}$-1	2hr		50%		50%
$dTAG^{V}$-1	2hr				100%
$dTAG^{V}$-1	2hr			40%	60%
$dTAG^{V}$-1	3hr		71%	29%	
$dTAG^{V}$-1	3hr		71%	29%	
$dTAG^{V}$-1	4hr	100%			
$dTAG^{V}$-1	4hr	67%	33%		

C

E7.5 embryos

dTAG	Duration	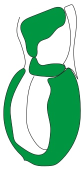	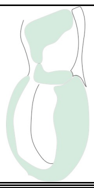	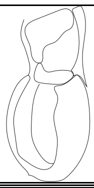
dTAG-13	2hr	100%		
dTAG-13	2hr	42%	42%	16%
$dTAG^{V}$-1	2hr		50%	50%
$dTAG^{V}$-1	2hr	57%	43%	
$dTAG^{V}$-1	2hr	60%		40%

Supplemental Figure S3: Table summarising the broad range of Eomes depletion in dTAG-13 or dTAGv-1 treated embryos *in utero*.

Results from individual litters after IP injection with dTAG-13 or $dTAG^V$-1 at E5.5 (A), E6.5 (B) or E7.5 (C). Indicated is the small molecule used, the duration of the treatment, and expression profile for the distinct stages for individual litters. (A) Eomes expression at E5.5 is restricted to the ExE and the EmVE and shows no change following dTAG-13 treatment (B) Expression at E6.5 is lost to varying degrees. There are 4 different classes of Eomes expression after treatment: Class I, Eomes is observed in all 3 Eomes expressing cell populations (ExE, EmVE and proximal posterior epiblast /PS); Class II, Eomes expression is lost in the EmVE but present in the ExE and proximal posterior epiblast /PS; Class III, Eomes is only evident in the proximal posterior epiblast /PS region; Class IV, little to no Eomes expression is observed. (C) At E7.5, Eomes expression after treatment corresponds to 3 categories, fully present, partially depleted or full depletion.

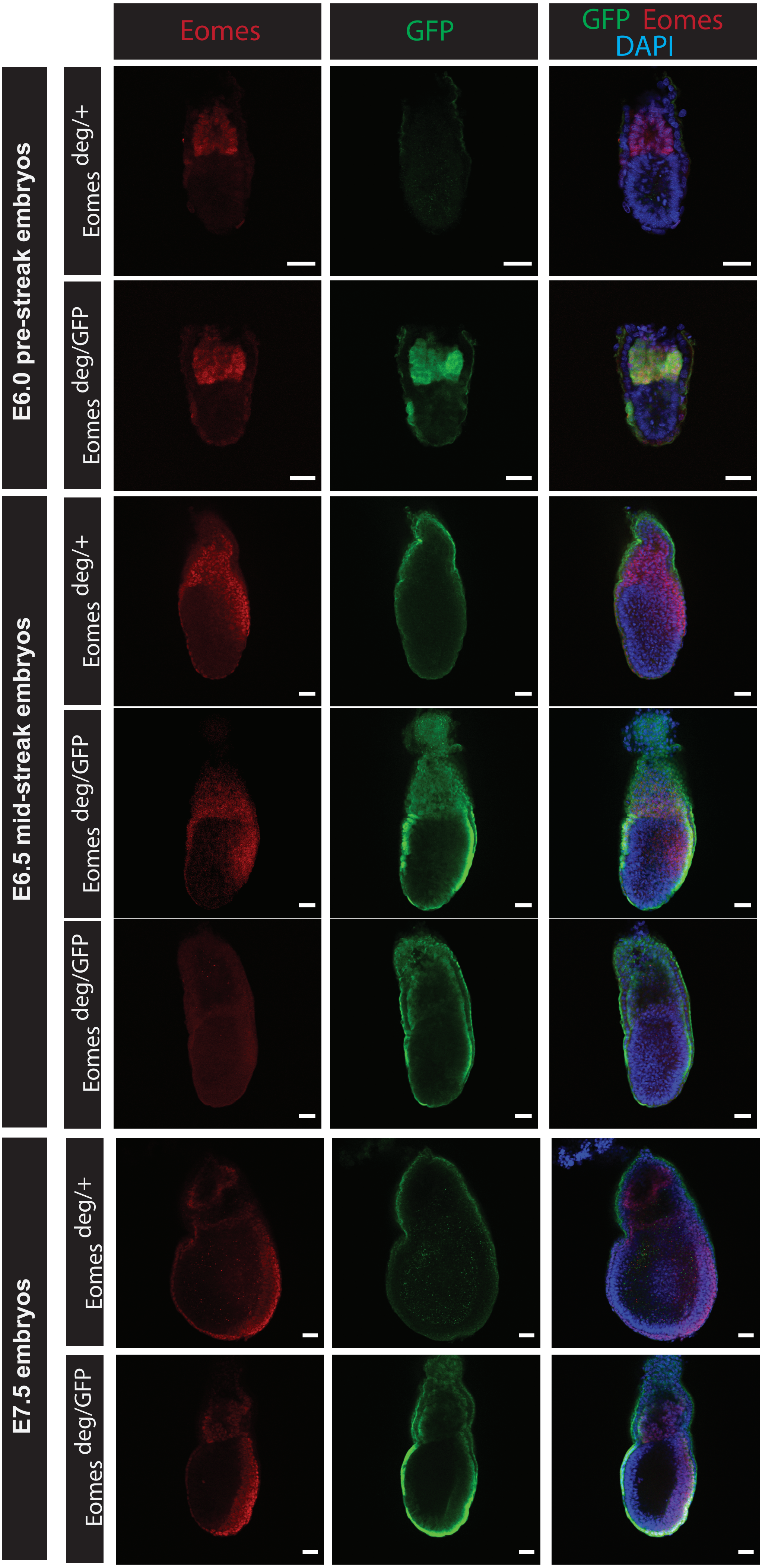

Eomes
GFP
GFP Eomes
DAPI
E6.0 pre-streak embryos
Eomes deg/+
Eomes deg/GFP
E6.5 mid-streak embryos
Eomes deg/+
Eomes deg/GFP
Eomes deg/GFP
E7.5 embryos
Eomes deg/+
Eomes deg/GFP

Supplemental Figure S4: dTAG-13 treatment of $Eomes^{deg/GFP}$ or $Eomes^{deg/+}$ embryos *in utero*.

Eomes (red) and GFP (green) staining of E6.0, E6.5 or E7.5 $Eomes^{deg/GFP}$ and $Eomes^{deg/+}$ embryos generated from $Eomes^{deg/deg}$ and $Eomes^{GFP/+}$ intercrosses, stained 2 hours post-injection with dTAG-13. Images representative of minimum 3 embryos. Nuclei are stained with DAPI (blue). Scale bars, 50 µm.

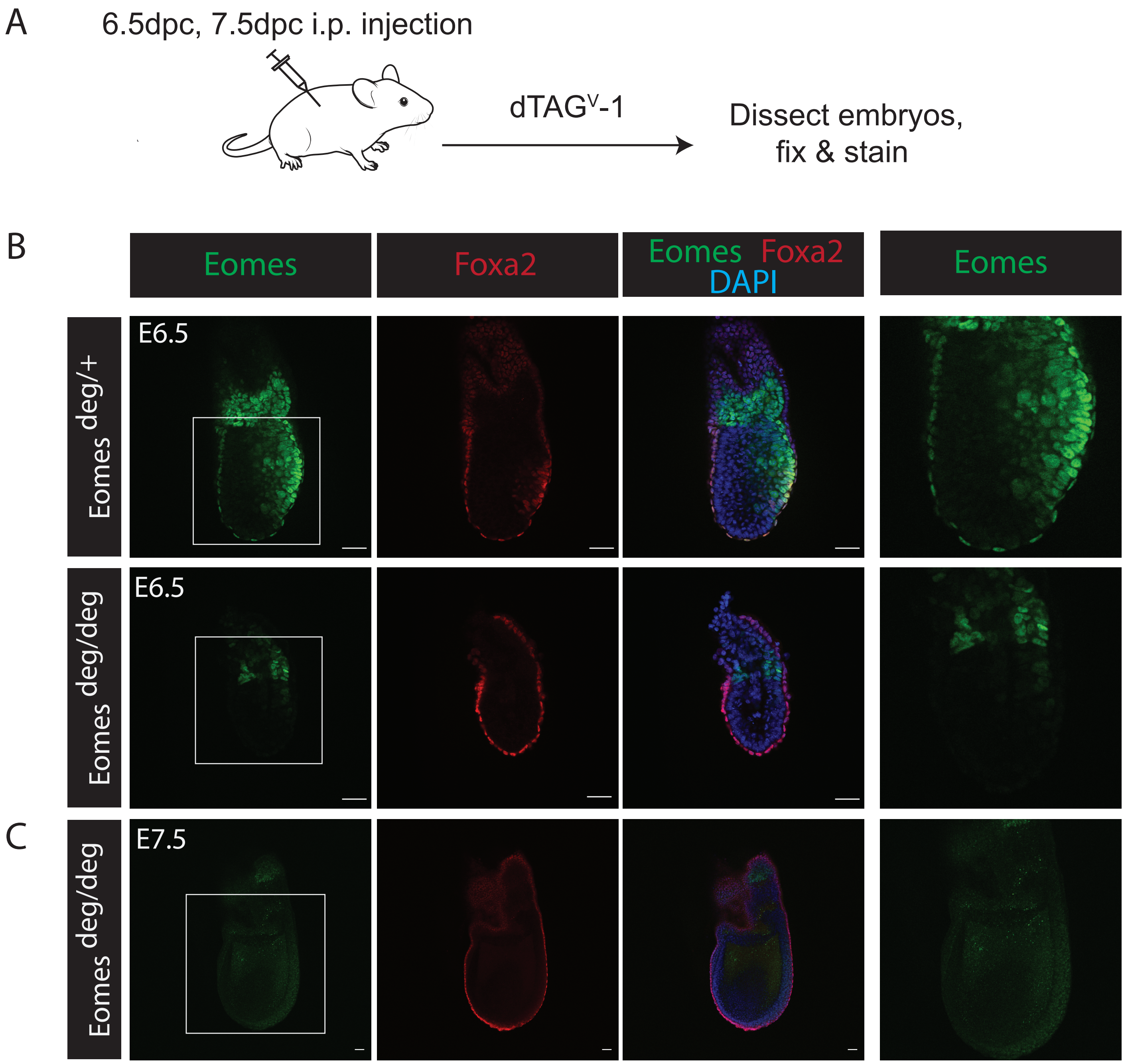

Supplemental Figure S5: Eomes expression is differentially reduced in a spatial pattern in Eomes$^{deg/deg}$ embryos after IP injection dTAG^{V}-1 at 6.5dpc and 7.5dpc.

(A) Schematic representation of the experimental protocol designed to test Eomes degradation *in utero* at 6.5dpc and 7.5dpc. (B&C) Immunofluorescence staining of E6.5 (B) and E7.5 (C) Eomes$^{deg/+}$ and Eomes$^{deg/deg}$ embryos recovered 2 hours (h) after pregnant females were injected IP with dTAG^{V}-1. White boxes indicate the magnified areas of Eomes expression (green) displayed in the right panels. Images representative of 9 embryos (E6.5) and 8 embryos (E7.5). Foxa2 staining (red) highlights VE and definitive endoderm cells. Nuclei are stained with DAPI (blue). Scale bars, 50 µm.

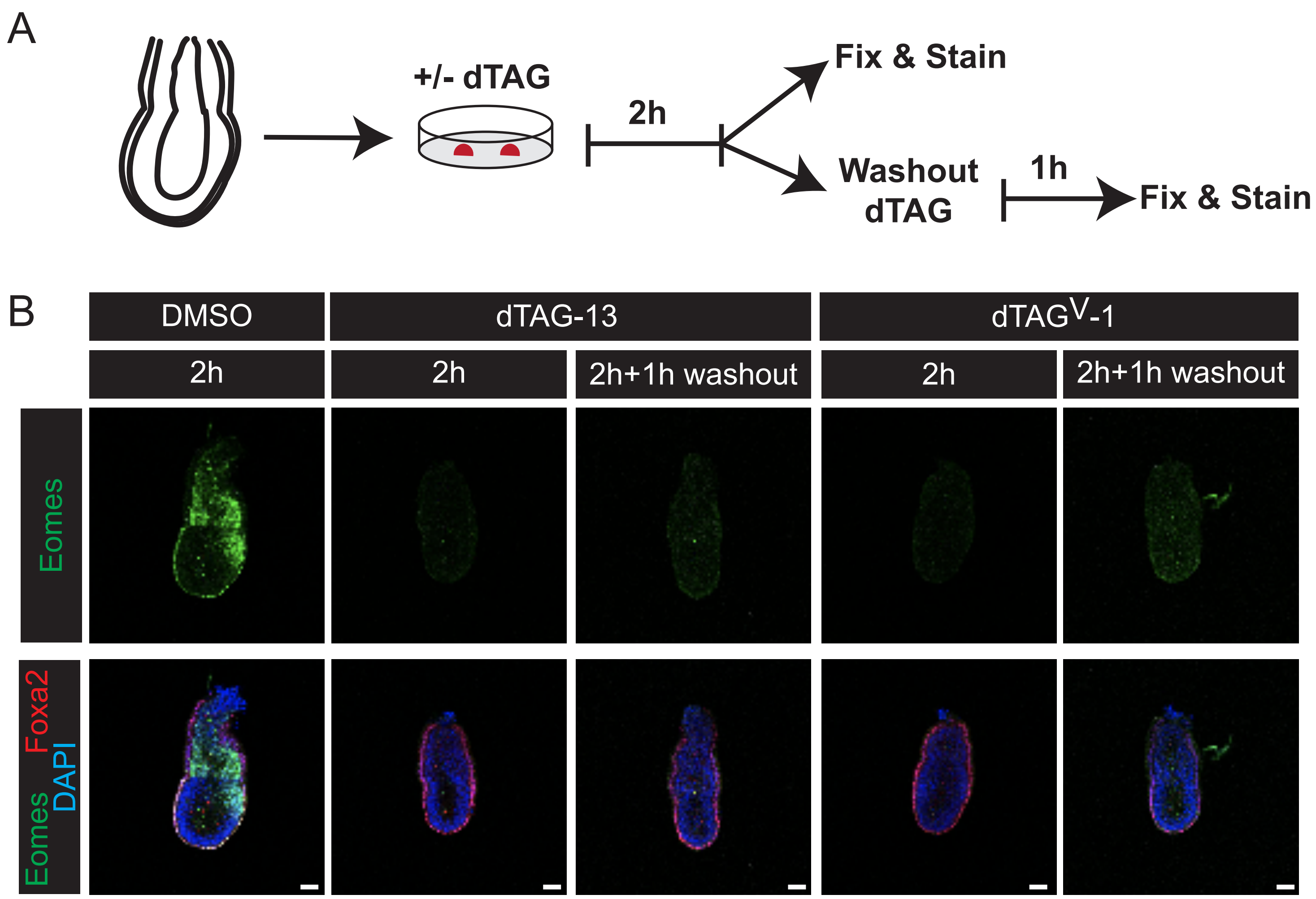

Supplemental Figure S6: *Ex vivo* treatment of Eomes$^{deg/deg}$ embryos with dTAG-13 or dTAG^{V}-1 and assessment of Eomes recovery after 1 hour washout

(A) Schematic representation of the experimental protocol for dTAG small molecule treatment and 1 hour (h) subsequent washout of E6.5 embryos. (B) Eomes (green) and Foxa2 (red) staining of DMSO, dTAG-13 or dTAG^{V}-1 treated E6.5 *ex vivo* cultured embryos fixed after 2 hours of treatment or washed out and cultured in the absence of dTAG small molecules for a further hour to assess recovery of Eomes expression. Nuclei are stained with DAPI (blue). Scale bars, 50 μm.

Table S1: Antibodies

**Reagent or Resource**	**Source**	**Identifier**	**Lot #**	**Dilution/ Concentration**	**Application**
Eomes/tbr2 rabbit polyclonal	Abcam	ab23345	GR3390354-1	1:200	Immunofluorescence
EOMES rat monoclonal (Dan11mag)	Invitrogen	14-4875-82	2288589	1:400	Immunofluorescence
Rabit anti-RFP	Rockland antibodies	600-401-379	30882	1:100	Immunofluorescence
Chicken anti-GFP	Abcam	ab13970	1018753-8	1:1000	Immunofluorescence
Human Otx2 goat polyclonal	R&D systems	AF1979	KNO0615111	1:100	Immunofluorescence
Human HNF-3 beta /FoxA2 goat polyclonal	R&D systems (biotechne)	AF2400	ULB1021081	1:100	Immunofluorescence
Anti-Nanog rabbit polyclonal	Abcam	ab80892	GR3223662-1	1:100	Immunofluorescence
Alexa Fluor 488 Donkey Anti-Rabbit IgG	Life Technologies	A21208	1932496	1:400	Immunofluorescence
Alexa Fluor 594 Donkey Anti-goat IgG	Molecular Probes	A11058	1445994	1:400	Immunofluorescence
Alexa Fluor 488 Goat anti-chicken IgG	Invitrogen	A11039	1637891	1:400	Immunofluorescence
AF488 α-mouse Eomes	Invitrogen	53-4875-82	2106734	1:1000	Flow cytometry
APC α-mouse Flk-1	Thermo Fisher	17-5821-81	2284224	1:40	Flow cytometry
PeCy7 α-mouse PdgfRa	Thermo Fisher	25-1401-82	4334654	1:160	Flow cytometry
Rabbit polyclonal α-β-tubulin	Cell Signaling	2146	9	1:2000	Western blot
Donkey α-rabbit IgG, HRP conjugated	Amersham	NA934V	321803	1:2000	Western blot
Goat α-rat IgG, HRP conjugated	Cell Signaling	7077	14	1:2000	Western blot

Table S2: Primers

Allele	Primer Name	Sequence	Primer Name	Sequence	Product
**Genotyping**					
Eomes+	AB_41_F2	TCTAGACTCCAGCGACTCCG	AB_41	TCTGGGCTGCTGTTTGCTTA	607bp
Eomesdeg	AB_40	CCCACAACGAGGACTACACT	AB_41	TCTGGGCTGCTGTTTGCTTA	515bp
Eomes wt	Eo_F	CCTTACTTCCTAAGGCACACCTGTAGC	Eo_WT_R	AGCTCAAACCGACTTCTTTCTGCTC	313bp
Eomes null	Eo_F	CCTTACTTCCTAAGGCACACCTGTAGC	Eo_Null_R	AATATGTCCTCAGGGAGATGATGACTC	456bp
Eomes wt2	GFP_F	AAGGAAAGGGGCACCTACAATC	GFP_R1	AGCACTCTCCAGCGAGTAGAAGTG	302bp
Eomes^{GFP}	GFP_F	AAGGAAAGGGGCACCTACAATC	GFP_R2	CAGATGAACTTCAGGGTCAGCTTG	373bp
**Screening**					
Eomes+	AB_38	GTGTACAACAGCGCTTGCAA	AB_41	TCTGGGCTGCTGTTTGCTTA	585bp
Eomesdeg	AB_38	GTGTACAACAGCGCTTGCAA	AB_41	TCTGGGCTGCTGTTTGCTTA	1728bp

Table S3: Reagents

Reagent or Resource	Source	Identifier	Lot #	Dilution/ Concentration	Application
ECL prime	Sigma-Aldrich	RPN2236		Manufacturer's instructions	Western blot
M2 medium	Sigma-Aldrich	M7167-100ML	SLCN7467		Cell Culture
0.25% Trypsin-EDTA (1X)	Gibco	25200-056	5997		Cell Culture
PBS	Gibco	14190-169	2430493		Cell Culture
Neurobasal medium	Gibco	21103049	2646164		Cell Culture
DMEM	Gibco	11960-044			Cell Culture
DMEM/F12	Gibco	11320033	2436794		Cell Culture
N2	Gibco	17502048	2313310	0.5x	Cell Culture
B27	Gibco	17504044	2090352	0.5x	Cell Culture
Pen/Strep	Gibco	15140-122	1825957	1%	Cell Culture
L-glutamine	Gibco	2430493	01724	1%	Cell Culture
β-mercaptoethanol	Sigma-Aldrich	M-7522	033K0080		Cell Culture
BSA Fraction V (7.5%)	Gibco	15260037	2517888	0.67%	Cell Culture
PD-0325091	University of Dundee, MRC-PPU unit	N/A	N/A	1µM	Cell Culture
CHIR99021	University of Dundee, MRC-PPU unit	N/A	N/A	3µM	Cell Culture
LIF	Millipore	ESG1107	8061495597	1000 U/ml	Cell Culture
TrypLE Express (1X)	Gibco	12605-010	2342307		Cell Culture
FBS	Gibco	10500-064	2232232H		Cell Culture
IMDM	Gibco	12440053	2581956		Cell Culture
PolyHEMA	Sigma	P3932-10G	SLBZ6865		Cell Culture
rhActA	R&D Systems	338-AC	BNV4118101	5 ng/ml	Cell Culture
rhVEGF	R&D Systems	293-VE	II6218092	5 ng/ml	Cell Culture
rhBMP4	R&D Systems	314-BP	BEM111810 1	10 ng/ml	Cell Culture
NEBuilder HiFi DNA Assembly Master Mix	New England Biolabs	E2621			Molecular Biology
NEB Stable cells	New England Biolabs	C3040H	10070108		Molecular Biology
Denaturing Gel-loading Buffer	Biodynamic s Laboratory	DS612	028BH08B		Molecular Biology

ZymocleanTM Gel RNA recovery kit	Zymo Research	R1011	ZRC204428		Molecular Biology
dTAG-13	Bio-Techne (R&D Systems)	6605/5	5A/268081	500nM	IF
dTAGv-1	Bio-Techne (R&D Systems)	6914/5	3A/268880	500nM	IF
DMSO	Sigma	D2650-100ML	RNBJ8032	500nM	IF
Vectashield	Vector Laboratories	H-1200	ZH1021	NA	IF
DAPI (10 mg/ml in water)	Biotium	40043	15D117-105958	1:5000	IF
Donkey serum	Sigma	D9663	SLBG0267	NA	IF
Kolliphor EL	Sigma-Aldrich	C5135-500G	102449680	10% v/v	IF
DAPI (1.0 mg/ml)	BD Pharmigen	564907	8012653	1:5000	Flow cytometry
Viability dye	Invitrogen	L34955	2329022	Manufacturer's instructions	Flow cytometry
Fixation/Permeabilization Concentrate	eBioscience	00-5123-43			Flow cytometry
Fixation/Permeabilization Diluent	eBioscience	00-5223-56			Flow cytometry
Permeabilization Buffer	eBioscience	00-8333-56			Flow cytometry
Nunc™ Lab-Tek™ II Chambered Coverglass	Thermo Scientific	155409	6041980		IF
BD 1ml Syringe Complete	BD Medical	305501	9154711		IF
BIOMAX XAR Film	Carestream	1651454			Western blot
EveryBlot blocking buffer	BioRad	12010020			Western blot
DCTM Protein Assay Kit I	BioRad	5000111			Western blot
Laemmli sample buffer	BioRad	1610747			Western blot
Mini-Protean® PAGE gel	BioRad	4561094			Western blot
